# Single-photon sensitive light-in-fight imaging

**DOI:** 10.1038/ncomms7021

**Published:** 2015-01-27

**Authors:** Genevieve Gariepy, Nikola Krstajić, Robert Henderson, Chunyong Li, Robert R. Thomson, Gerald S. Buller, Barmak Heshmat, Ramesh Raskar, Jonathan Leach, Daniele Faccio

**Affiliations:** 1 Institute of Photonics and Quantum Sciences, Heriot-Watt University, David Brewster Building, Edinburgh EH14 4AS, UK; 2 Institute for Micro and Nano Systems, University of Edinburgh, Alexander Crum Brown Road, Edinburgh EH9 3FF, UK; 3 Queen's Medical Research Institute, University of Edinburgh, 47 Little France Crescent, Edinburgh EH16 4TJ, UK; 4 Camera Culture, MIT Media Lab, MIT, 75 Amherst Street, E14-474, Cambridge, Massachusetts 02139, USA

## Abstract

The ability to record images with extreme temporal resolution enables a diverse range of applications, such as fluorescence lifetime imaging, time-of-flight depth imaging and characterization of ultrafast processes. Recently, ultrafast imaging schemes have emerged, which require either long acquisition times or raster scanning and have a requirement for sufficient signal that can only be achieved when light is reflected off an object or diffused by a strongly scattering medium. Here we present a demonstration of the potential of single-photon detector arrays for visualization and rapid characterization of events evolving on picosecond time scales. The single-photon sensitivity, temporal resolution and full-field imaging capability enables the observation of light-in-flight in air, as well as the measurement of laser-induced plasma formation and dynamics in its natural environment. The extreme sensitivity and short acquisition times pave the way for real-time imaging of ultrafast processes or visualization and tracking of objects hidden from view.

## Abstract (web summary)

Ultrafast imaging schemes can enable a diverse range of applications but require long acquisition times or raster scanning. Here, Gariepy *et al*. demonstrate visualization and rapid characterization of light-in-flight and laser-induced plasma formation using single-photon detector arrays.

## Graphical abstract

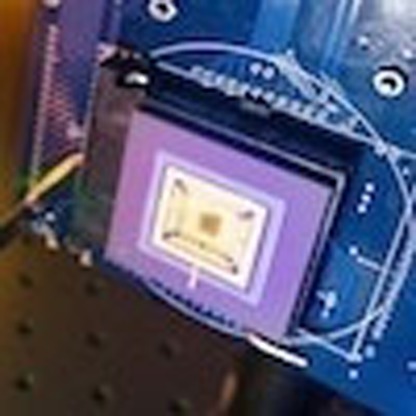

Light was first captured in its flight by Abramson in 1978 (ref. 1), who used a holographic technique to record the wavefront of a pulse propagating and being scattered by a white-painted screen placed in its path. This high-speed recording technique allowed the dynamic observation of light phenomena like reflection, interference and focusing that are normally observed statically^2^,^3^. More recently, light-in-flight holography has been performed in a scattering medium rather than using a reflective screen^4^,^5^. Light can also be captured in motion in a scattering medium using a streak camera that has picosecond temporal resolution, thus removing the need for interferometry and coherent illumination but requires additional hardware to raster scan the two-dimensional (2D) scene, which increases the acquisition time to hours^6^,^7^. A few other techniques possess the temporal resolution to observe light in motion as it illuminates a scene, such as photonic mixer devices based on modulated illumination, albeit with a temporal resolution limited to a few nanoseconds^8^. Alternatively, time-encoded amplified imaging can record images at the repetition rate of a laser by exploiting wavelength-encoded illumination of a scene and amplified detection through a dispersive fibre, albeit with 160 ns temporal and spatial resolution^9^. Recent studies based on computer tomography using data from multiple probe pulses enabled reconstruction of picosecond pulse propagation phenomena in condensed media^10^. The current challenge is to simplify data acquisition and reduce acquisition times by achieving full imaging capability and low-light sensitivity while maintaining temporal resolution in the picosecond regime.

Here we address this challenge and provide an imaging solution that simultaneously acquires spatial and temporal information (*x*, *y* and *t*). The technology we use is based upon a two-dimensional Silicon CMOS array of single-photon avalanche diode (SPAD) detectors with each individual pixel operated in time-correlated single-photon counting (TCSPC) mode^11^,^12^,^13^,^14^. Individual SPAD detectors are increasingly used throughout the field of optics; in particular, their high temporal resolution has made them useful for single-photon time-of-flight measurements^15^,^16^, fluorescence lifetime imaging^14^ and photon counting^17^. Recent advances in electronics have enabled the development of arrays of SPADs, giving the capability to perform light-in-flight measurements. In these experiments, we use a 32 × 32-pixel array that has single-photon sensitivity and acquires time information with a resolution of 67 ps, which provides the ability to freeze the motion of light with a blurring of only a few centimeters^18^.

## Results

### Experimental setup

**Figure 1:**
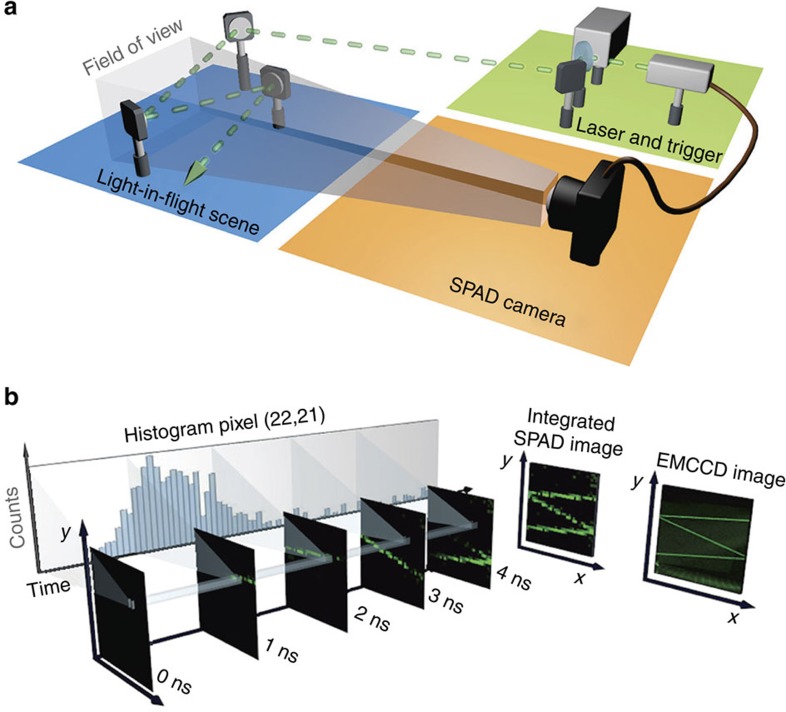
Light-in-flight measurement and data cube. (**a**) A laser pulse is reflecting off multiple mirrors, passing three times across the field of view of the SPAD camera (35 × 35 cm^2^). The same laser is used to create a trigger sent to the camera. The SPAD camera collects scattered photons from the laser pulse. The field of view does not contain the mirrors because the scattered light coming from the mirror surfaces is much more intense than the Rayleigh-scattered light during propagation. (**b**) The histogram indicates the time of arrival of the laser pulse as measured by pixel (22, 21). The time frames, shown at 0, 1, 2, 3 and 4 ns, show the evolution of the pulse in time as it propagates across the scene. The integration of all frames gives the total path followed by the light, similarly to what can be acquired by an EMCCD camera at maximum gain for an exposure time of 7 s.

The setup for our experiment is illustrated in Fig. 1. The scene is imaged onto the SPAD array using a fisheye lens. Laser pulses propagate at 4 kHz from right to left across the field of view and are reflected by two mirrors. Light from the laser pulses is scattered by air molecules and then detected by the SPAD camera.

The SPAD camera is operated in TCSPC mode: every pixel has its own picosecond timer. The timer is started by the detection of a single photon, and stopped by the arrival of a periodic trigger, which is derived from the laser pulses. The time between start and stop is recorded, and a histogram of photon arrival times builds up as a number of optical pulses successively propagate through the setup. The camera is connected directly to a computer and we acquire data for the histograms during a 10-min period—this corresponds to 2 million identical pulses propagating through the setup. Use of a higher repetition rate laser (MHz) would result in the acquisition time being reduced to less than 1 s.

Figure 1 gives a schematic representation of the data cube (*x*, *y* and *t*) acquired by the SPAD array. The histogram in Fig. 1 shows a 5-ns section for one of the pixels in the array: considering all of the *x*, *y* locations at one particular time *t* gives a snapshot of the spatial position of the laser pulse as shown for example in the individual time frames in Fig. 1. Integrating all time frames gives a static representation of the path followed by the light, similar to what is captured with an EMCCD camera, also shown in Fig. 1.

### Data processing

**Figure 2:**
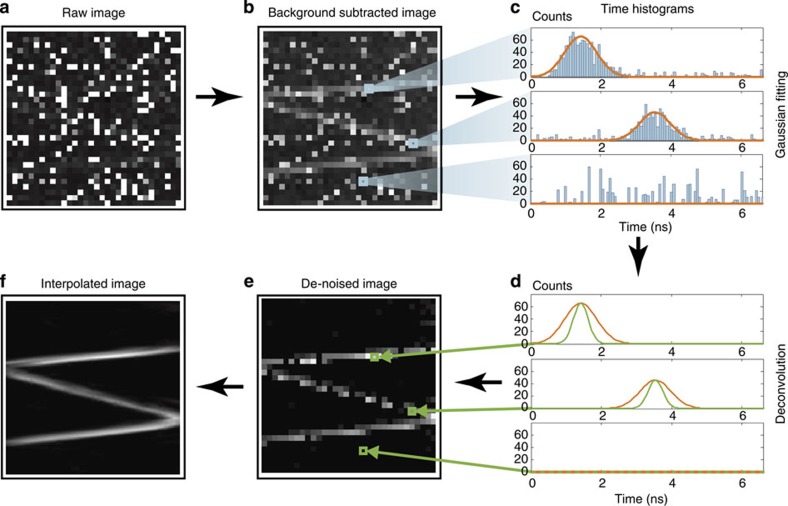
Data processing. (**a**) To process the acquired data, we first subtract a background acquired by the SPAD camera without the laser beam propagating across its field of view. It allows us to see the path, but the image (**b**) is still very noisy. (**c**) To get a clearer image, we fit a Gaussian to every histogram. If the Gaussian is much wider than we expect, or smaller than the pulse duration, we set the histogram to zero. (**d**) We then deconvolve the fitted Gaussians down to a 500-ps Gaussian (**e**). (**f**) Finally, to improve the resolution of our frames, we interpolate between the centre of mass of two adjacent lines/columns.

The unique features of the three-dimensional data allow efficient data processing and noise suppression in both the temporal and spatial dimensions. The image processing algorithm is schematically illustrated in Fig. 2 that shows the time-integrated image after each step of the data processing. Starting with the raw data, we first subtract the background data cube acquired in the same conditions, but without the laser propagating through the scene. Second, every time histogram is fitted with a Gaussian. For pixels containing signal from the laser pulse, this fitting removes what we could call the ‘temporal noise’, that is, the noise in the time bins with no signal from the laser pulse. Moreover, some spatial pixels are never illuminated by the laser pulse scatterings and contain only noise and background light and are thus set to zero. We therefore use the time information contained in each pixel to improve the spatial quality of our data. Third, we deconvolve the instrument impulse response function (largely due to electronic jitter and measured by uniformly illuminating the camera with a 100-fs laser pulse) from the Gaussian fits. This results in histograms with a full-width at half-maximum of ∼500 ps FWHM that is consistent with an independent measurement of the laser pulse duration. Finally, we increase the spatial quality of the images by a standard linear re-interpolation, frame by frame, between each line and column of the 32 × 32 pixel array as in Fig. 2. We interpolate in a way that connects the centre of mass of each line and column, in order to achieve a final spatial 310 × 310 pixel count. Summarizing, the data processing essentially relies on a three-stage process composed of noise removal, temporal deconvolution and re-interpolation: this process does not require any *a priori* knowledge of the scene content itself and is therefore inherently robust. The re-interpolation process is a very basic nearest-neighbour interpolation: no information is lost in this process and, naturally, no information is gained either. More importantly, all quantitative data analysis (for example, the plasma decay times as discussed below), is always performed on the raw (not interpolated) data.

### Light propagation in air

**Figure 3:**
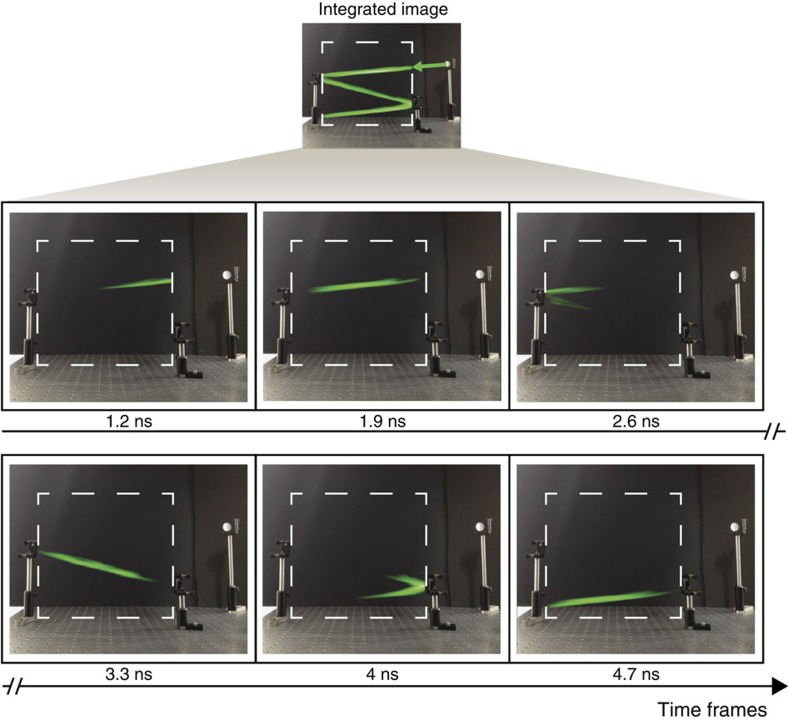
Frames of the light-in-flight movie. In these frames, we see a pulse of light propagating between three mirrors. The laser first hits the mirror on the right and is directed towards the field of view of the SPAD camera, as indicated by the green arrow on the integrated image. The FOV is represented by dashed rectangles and corresponds to a 35 × 35 cm^2^ region. In the first and second frames, we show the laser pulse entering the FOV. In the second, third and fourth frames, we see the light being reflected by the mirrors, before exiting the FOV in the last frame.

The final result is shown in Fig. 3 where we show the evolution of the light pulse propagating through air. The figure shows selected time frames acquired by the camera, each separated by 0.7 ns and overlaid on a photograph (taken with a commercial DSLR camera) of the setup. The field of view of the SPAD camera is represented by dashed lines on each frame. In the first frame, the laser pulse has just entered the field of view. We then see the pulse propagate and reflecting off the two mirrors in the subsequent frames, before finally leaving the field of view. A video of the full evolution of the laser pulse propagation is provided as Supplementary Video 1. The scene that we observed, that is, the pulse reflecting between the three mirrors, happens within a few nanoseconds, only a fraction of the 69 ns time range of the camera. During that time, the 500 ps pulse, which is 15-cm long, travels 67 ps or 2 cm between each frame of the movie. The figure (and video) clearly demonstrates that we are able to capture the full dynamic of light propagation in air.

In these measurements, the average count rate from the detector is found to be of the order of 0.0005 detected events per laser pulse, per pixel. Even allowing for the relatively low fill-factor and single-photon detection efficiency, this implies a photon flux of the order of 0.2 photons per pulse, per full pixel area. The combination of single-photon sensitivity and picosecond temporal resolution make it very difficult for detector technologies such as intensified CCD^19^ to achieve the results shown.

### Plasma dynamics

**Figure 4:**
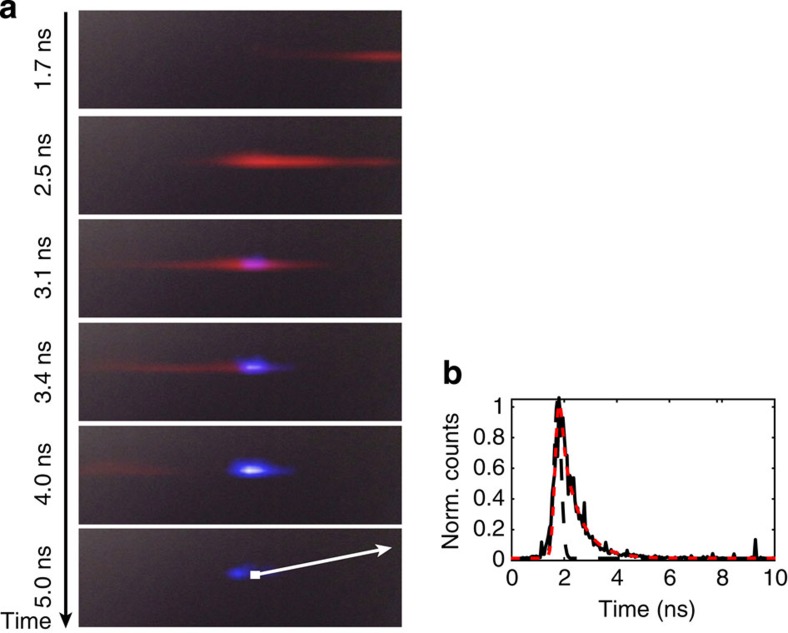
Plasma evolution recorded by light-in-flight. (**a**) In these frames, the femtosecond pulse is shown in red, and the plasma fluorescence in blue. The plasma is created as soon as the leading edge of the pulse passes through focus. The laser pulse is then scattered by the plasma, which can be seen in the second frame. As a large portion of the beam is scattered or absorbed at the focus by the plasma, the exiting beam is weaker, as we observe in the third to fifth frames. We also see the plasma being formed at focus when the laser pulse is focused, then evolving and decaying after the pulse has passed. A full video is provided as Supplementary Video 2. (**b**) The graph shows the temporal histogram of the plasma fluorescence (solid black curve, normalized photon counts, raw data) corresponding to the region indicated with a white square in the last frame. The dashed line is the detector impulse response function, the dotted red curve is an exponential decay fit with a 600-ps decay constant.

We also captured the dynamics of a more complex light phenomenon that is still the object of intense research studies for various applications: the formation of a weakly ionized plasma filament by a femtosecond laser pulse^20^. Laser pulses with 90 fs, 12 mJ (Amplitude Technologies), 785 nm wavelength at a 100-Hz repetition rate were loosely focused with a 50-cm lens to create a weak and tenuous plasma filament approximately 5 cm long, in the field of view of the SPAD camera. The weakly ionized plasma filament emits a faint fluorescence signal, largely in the 300–440 nm wavelength range^21^. The data were acquired for 2 min in two different spectral ranges using coloured filters: in red, to isolate the propagation of the laser pulse, and in blue, to isolate the plasma fluorescence. Figure 4 shows the scene for selected time frames (a full video is available as Supplementary Video 2). We first observe the femtosecond pulse (red) coming into the field of view and becoming brighter in the second frame as the pulse is scattered by the plasma created by its leading edge. The beam then moves out of the field of view, with a reduced intensity because of scattering from the self-induced plasma. We can also see the plasma (blue) being formed and still fluorescing well after the beam has passed. The time histograms (an example is also shown in Fig. 4) reveal an exponential decay with a lifetime of approximately 600 ps, consistent with previous measurements^20^. It is important to note that the technique described here allows to characterize the plasma dynamics without the need to introduce any additional scattering agents, which would severely alter the plasma formation process itself.

## Discussion

Our measurements open new possibilities for measuring ultrafast temporal dynamics within extremely low-intensity events. Remarkably, we are now able to observe light-in-flight at the sub-nanosecond scale in air where scattering occurs only from the ambient gas molecules. We underline that the SPAD camera not only allows to record a scene dynamically on very short-time scales, but it also provides an additional opportunity for noise reduction by exploiting the temporal dimension. Other applications are viable where the ability to record low photon numbers with high temporal resolution will enable viewing objects that are hidden from view, for example, looking around corners^22^,^23^,^24^ or through walls^24^,^25^,^26^. The single-photon sensitivity of our ultrafast imaging method enables direct spatially resolved measurements of dynamic quantum phenomena^19^, and also very weak scattering phenomena such as air-born acousto-optic effects^27^.

## Methods

### Details of experimental setup

The fisheye lens of 12-mm focal length provides a 35-cm wide field of view at the chosen object distance of 1.8 m. The laser used to observe light reflecting off mirrors is a 532-nm wavelength microchip laser (Teem Photonics STG 03E), which emits 500 ps duration pulses of 3.5 μJ energy at 4 kHz repetition rate. The laser polarization is set to vertical to maximize scattering in the direction of the camera. Experiments repeated in a pure nitrogen environment (data not shown) gave identical light intensities on the camera, thus verifying that the scattering is from air molecules and not from any possible dust particles that may be present in a standard atmospheric environment.

### Time-correlated single-photon counting

The TCSPC timer of the SPAD array is operated in reverse mode, that is, it is started by the detection of a single photon, and stopped by the arrival of a periodic trigger. The trigger is derived from detection of a reflection of the laser pulse with an optical constant fraction discriminator (Becker & Hickl OCF-401). The time range of the histogram acquired in TCSCP mode is 68.6 ns with a bin-width of 67 ps (determined by the 10 bit acquisition electronics), and the median dark count rate of the pixels is 50 counts per second. In the experiment, the SPAD camera detects an average of 0.0005 photon per pulse per pixel, thus operating in the photon-starved regime as required for TCSPC.

## Additional information

**How to cite this article**: Gariepy, G. *et al*. Single-photon sensitive light-in-fight imaging. *Nat. Commun.* 6:6021 doi: 10.1038/ncomms7021 (2015).

## Supplementary Material

Supplementary Video 1 Light propagation in air

Supplementary Video 2 Formation and evolution of laser-induced plasma
